# Automated Pipeline to Generate Anatomically Accurate Patient-Specific Biomechanical Models of Healthy and Pathological FSUs

**DOI:** 10.3389/fbioe.2021.636953

**Published:** 2021-01-28

**Authors:** Sebastiano Caprara, Fabio Carrillo, Jess G. Snedeker, Mazda Farshad, Marco Senteler

**Affiliations:** ^1^Department of Orthopedics, University Hospital Balgrist, University of Zurich, Zurich, Switzerland; ^2^Institute for Biomechanics, Swiss Federal Institute of Technology (ETH), Zurich, Switzerland; ^3^Research in Orthopedic Computer Science, University Hospital Balgrist, Zurich, Switzerland

**Keywords:** deep learning, patient-specific 3D model, FE analysis, surgical planning and simulation, spine-pathology

## Abstract

State-of-the-art preoperative biomechanical analysis for the planning of spinal surgery not only requires the generation of three-dimensional patient-specific models but also the accurate biomechanical representation of vertebral joints. The benefits offered by computational models suitable for such purposes are still outweighed by the time and effort required for their generation, thus compromising their applicability in a clinical environment. In this work, we aim to ease the integration of computerized methods into patient-specific planning of spinal surgery. We present the first pipeline combining deep learning and finite element methods that allows a completely automated model generation of functional spine units (FSUs) of the lumbar spine for patient-specific FE simulations (FEBio). The pipeline consists of three steps: (a) multiclass segmentation of cropped 3D CT images containing lumbar vertebrae using the DenseVNet network, (b) automatic landmark-based mesh fitting of statistical shape models onto 3D semantic segmented meshes of the vertebral models, and (c) automatic generation of patient-specific FE models of lumbar segments for the simulation of flexion-extension, lateral bending, and axial rotation movements. The automatic segmentation of FSUs was evaluated against the gold standard (manual segmentation) using 10-fold cross-validation. The obtained Dice coefficient was 93.7% on average, with a mean surface distance of 0.88 mm and a mean Hausdorff distance of 11.16 mm (*N* = 150). Automatic generation of finite element models to simulate the range of motion (ROM) was successfully performed for five healthy and five pathological FSUs. The results of the simulations were evaluated against the literature and showed comparable ROMs in both healthy and pathological cases, including the alteration of ROM typically observed in severely degenerated FSUs. The major intent of this work is to automate the creation of anatomically accurate patient-specific models by a single pipeline allowing functional modeling of spinal motion in healthy and pathological FSUs. Our approach reduces manual efforts to a minimum and the execution of the entire pipeline including simulations takes approximately 2 h. The automation, time-efficiency and robustness level of the pipeline represents a first step toward its clinical integration.

## Introduction

Patient-specific three-dimensional (3D) models are essential in computer-assisted surgical procedures. In spine surgery, computer-assisted techniques have been increasingly used in various stages of surgical planning and/or execution, e.g., to determine the optimal trajectory for the insertion of pedicle screws (Goerres et al., 2017; Knez et al., 2019; Mischler et al., 2020), but also to improve surgical navigation and allow an improved execution of the surgical plan (Liebmann et al., 2020; Müller et al., 2020). Biomechanical models can provide information on the preoperative pathological condition such as degenerative changes and their biomechanical consequences (Du et al., 2016; Cai et al., 2020). Patient-specific simulations can be used to analyze the effects of different surgical procedures on specific structures and pathologies. Potentially, the surgical plan can be improved based on the simulation output (Jiang and Li, 2019; Zhou and Willing, 2020). If included in the clinical workflow, such preoperative computational biomechanical analysis, in combination with precise intraoperative navigation, may improve patient outcomes. Finite element (FE) simulations of the lumbar spine have been employed in clinical applications to identify biomechanical parameters (Bernakiewicz and Viceconti, 2002; Little and Adam, 2012), evaluate surgical procedures, and analyze implants, e.g., for spinal fusion (Zhang et al., 2018) or total disc arthroplasty (Zhou and Willing, 2020). Such analyses may provide information on the expected bone and implant loads before surgery (Erbulut et al., 2015; Más et al., 2017; Özmen and Günay, 2019; Panico et al., 2020). Moreover, the biomechanical analysis of pathological spinal segments has the potential to provide indications on the degeneration process of intervertebral disc (IVD) and facet joints (FJs) (Rohlmann et al., 2006; Bashkuev et al., 2020; Cai et al., 2020), thus helping in the analysis of postoperative complications such as the development of adjacent segment degeneration (Li et al., 2015; Zhou and Willing, 2020). IVD and FJ degenerations alter the load transfer in the spine and are commonly associated with low back pain (Uçar et al., 2019; Bashkuev et al., 2020). Nevertheless, current standard clinical procedures lack the ability to perform such patient-specific biomechanical analysis on a daily basis, which hinders the possibility for optimizing the surgical plan.

One of the major challenges for inclusion of such methods in the clinical practice is the effort required to create patient-specific functional models from medical images. It includes several manual steps and is time-consuming even for experienced professionals (Sarkalkan et al., 2014; Campbell and Petrella, 2015). The time needed for the creation of patient-specific finite element (FE) models has rarely been reported, although critical for integrating biomechanical simulations in a clinical environment (Zadpoor and Weinans, 2015). To the best of our knowledge there is no complete automated pipeline for anatomically accurate FE simulations of the lumbar spine based on 3D CT images. A lot of work has been done on parametric FE models (Galbusera et al., 2008; Bashkuev et al., 2018, 2020; Lavecchia et al., 2018; Nikkhoo et al., 2020; Zhou and Willing, 2020) or a combination of statistical and FE models (Bonaretti et al., 2011, 2014; Rao et al., 2013; Campbell and Petrella, 2016). However, those models either neglect important patient-specific structures or their generation involve high amounts of manual work requiring certain types of operator expertise. Although efforts have been made to automate the generation of FE models of the healthy spine (Bah et al., 2009; Campbell and Petrella, 2015), hitherto this process has never been combined with deep learning-based segmentation methods and has not yet been applied to pathological cases. Nowadays, deep learning methods are employed in medical research to analyze images, extract structural information, and to localize and segment 3D structures (Glocker et al., 2012; Korez et al., 2015; Roth et al., 2016; Lessmann et al., 2019). They provide fast results in an automated fashion, with an accuracy comparable to those from manual human processing (Nikolov et al., 2018). Integrating these methods into the creation of patient-specific biomechanical models could drastically accelerate the process and enable a complete automated framework, which in turn would allow clinical routine applications of computational preoperative planning using 3D models (Zadpoor and Weinans, 2015). In previous studies, deformable models were added to a 3D convolutional neural network (CNN) (Korez et al., 2015) to perform segmentation of vertebral bodies from 3D magnetic resonance spine images, However, the method has not yet been applied to FE modeling. It has been also shown how the automatic creation of FE models could benefit from using a mesh-based registration method (Bonaretti et al., 2014).

**FIGURE 1:**
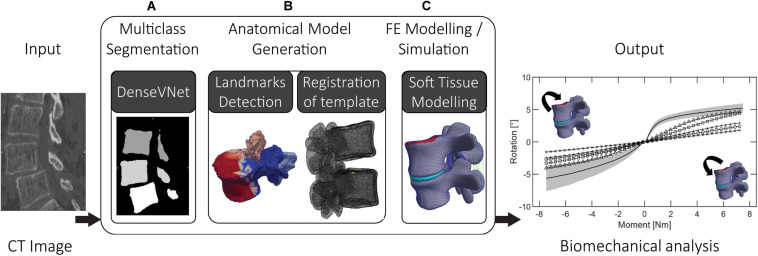
The three steps of the proposed pipeline: **(A)** multiclass segmentation of the CT image dataset, **(B)** semantic segmentation and non-rigid registration of template model, **(C)** FE modeling and simulation, involving complementing the anatomical models with soft tissues (ligaments, IVD) using automatic landmarks detection.

In this work, we propose a combination of deep learning, statistical, and FE methods on lumbar 3D CT images to generate anatomically accurate patient-specific FE models of FSUs. A biomechanical investigation of spinal segments may be highly clinically relevant, hence our main aim is the automation of the complete workflow (Figure 1). By saving time and reducing manual interaction for modeling and simulation, the main intent of the presented pipeline is a step toward a seamless clinical integration of such models. The goals are the ability to perform state-of-the-art segmentation of pathological (degenerative) lumbar spine’s segments and the execution of FE simulations with reasonable results. Using clinically available dataset, the implemented pipeline should provide a basis for further developments toward the integration of patient-specific modeling in clinical planning of spinal surgery. The automation is achieved by the integration of state-of-the-art deep learning methods and a novel interface to FE modeling. The outputs are anatomically accurate patient-specific biomechanical models and results of FE simulations.

## Materials and Methods

We have combined multiple deep learning methods using two 3D CNNs allowing the automated segmentation of lumbar vertebrae and identification of corresponding point sets on the vertebral meshes. Multiple training datasets were prepared and used to train the different networks needed for the segmentation of the 3D CT images and for the identification of anatomical regions on vertebral models (Figures 1A,B). We re-trained the DenseVNet segmentation network presented in Gibson et al. (2018a) within the NiftyNet platform (Gibson et al., 2018b). The correspondence of anatomical points and regions between the segmentation and the template model is achieved by an automated identification of anatomical landmarks on the vertebral surfaces through semantic segmentation. This step acts as an interface to perform non-rigid fitting of the template SSMs and improves the following non-rigid registration results (Clogenson et al., 2015). The method for landmarks identification employed feature steered graph convolutions (FeaStNet) described in Verma et al. (2018). Meticulously prepared reference meshes of the SSMs were a requirement to enable automatic identification of soft tissue insertion points and surfaces. Subsequently, a functional patient specific FE model is automatically created for different lumbar segments based on deformable template models. The goal of the resulting pipeline is to eliminate the time-consuming procedure of preparing FE models (Bah et al., 2009; Taylor and Prendergast, 2015; Wu et al., 2019).

Some of the methods implemented and combined in this work exist as individual implementations. Major effort was spent for their combination and integration into a single pipeline, and developing required interfaces. The correspondence property of the registered template meshes was crucial for the automatic creation of the FE models, e.g., for healthy and degenerated discs (Figure 1C). The exclusion of manual steps may result in more reliable and robust pipelines. Furthermore, the ability to biomechanically analyze pathological cases in an automated fashion seems highly relevant for computational surgery planning in an efficient clinical workflow.

### Multiclass Segmentation

#### Training Dataset

The preoperative clinical 3D CT images were selected from a larger dataset by exclusion of severe pathological vertebrae (ethics approval ID: BASEC: 2019-00698). The resulting dataset contains 52 3D CT images all acquired at Balgrist University Hospital between 2014 and 2019. The original field of view (FoV) was manually and systematically reduced by cropping the 3D images to contain the lumbar spine from level L2 until the sacrum. Final 3D images include 3 complete lumbar vertebrae (L5, L4, and L3). The resulting FoVs covered a range of 224–390 voxels (87–117 mm) transversely, and 107–402 voxels (107–141 mm) in the inferior-superior direction. Each cropped 3D image was manually segmented by a single trained radiologist to minimize variability, using the software Mimics 19.0 (Materialise Inc., Leuven, Belgium). Available region-growing and thresholding algorithms were used to generate 3D masks of the original 3D CT images. Distinctive labels were assigned to vertebrae of different levels. Similarly, the output of the trained network will not only contain the segmentation, but also a label discerning between vertebral levels. By cropping the images, we eliminated the class imbalance problem that could otherwise lead to a biased network (Hesamian et al., 2019), which occurs when the majority of the imaging dataset is occupied by background. After cropping, the 3D images were pre-processed by applying a histogram matching transformation (Woods and Gonzales, 1981), equalizing histograms with the reference histograms of an arbitrarily chosen CT image. This preprocessing step allowed the normalization of the intensity within the dataset and is easily applicable to any new image.

#### Training Process

**FIGURE 2:**
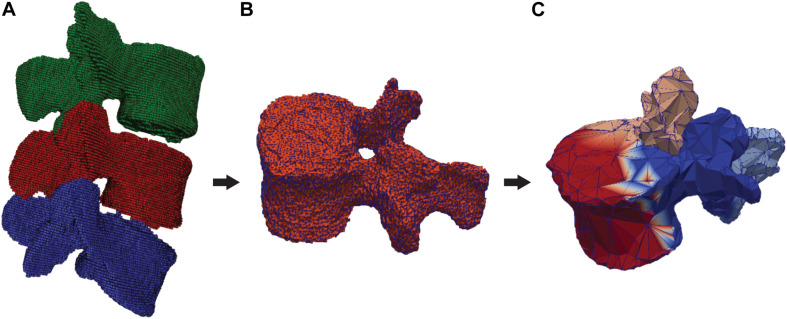
Segmented and labeled vertebrae and division in semantic parts. **(A)** DenseVNet output (L3: green, L4: red, L5: blue), **(B)** 3D segmentation of one vertebra (L4), **(C)** Output of the semantic segmentation of four regions of a vertebra (front: red, left: blue, right: pink, back: azure).

The DenseVNet network was re-trained using NiftyNet framework (Gibson et al., 2018b) on the 52 3D CT images. This network was originally developed to perform multiclass segmentation of different organs in abdominal CT and offers the possibility to perform segmentation of different structures with improved boundary accuracy. This peculiarity offers an advantage for the definition of facet joints’ boundaries between adjacent vertebral bones, particularly critical for the definition of contact surfaces for the FE analysis. The platform NiftyNet was explicitly designed for medical image analysis and it includes the DenseVNet network structure. We included the computation of the Dice-hinge coefficient (DC) losses for the segmentation of each vertebra as described in Gibson et al. (2018a) in the NiftyNet library. This loss function was chosen as it proved promising generalization properties thanks to the adapted weights for classes with low dice scores but significant gradients during training. As the training dataset included images of different dimensions and resolutions, the input volumes were resampled to a voxel dimension of [0.39, 0.39, 0.5]. The training was run for 4,000 iterations using the Adam optimizer with ε = 0.001 (Gibson et al., 2018a) on a Quadro P6000 GPU (NVIDIA Corporation, Los Alamitos, CA) and took 30 h. The output of the trained network is a 3D mask with four different classes, three for the vertebrae, and one for the background (Figure 1A). Using the different labels, each vertebral mask was post-processed by removing incorrectly segmented isolated regions with a significantly smaller area compared to the segmented vertebrae. Finally, three segmented models were generated, one for each vertebral level (Figure 2A), by performing three different triangulations in MATLAB R2019a (The MathWorks, Inc., United States) using the GIBBON package (Moerman, 2018).

### Anatomical Model Generation

#### Semantic Segmentation of Segmented Vertebrae

##### Training dataset

**FIGURE 3:**
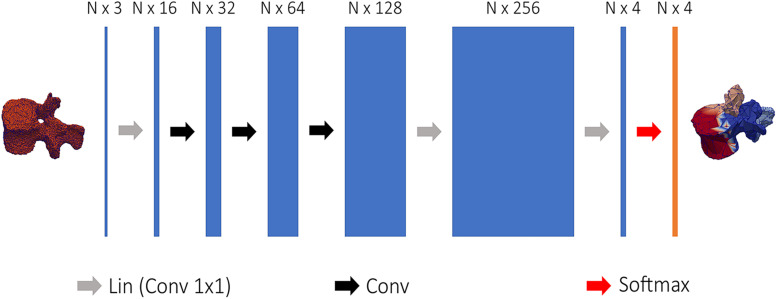
Structure of the network used to semantically segment vertebral models. 3D coordinates of the vertebral point cloud are input to the first linear convolution layer, changing the input feature dimensions. The following layers are the feature steered graph convolutions preceding 1 × 1 convolution to N × 4 logits, used to soft-assign each point of the point cloud to one of the four classes.

The ground truth images used for the training of the DenseVNet network were used to prepare 3D meshes of single vertebral structures. The segmented masks were used to export 3D models of individual vertebrae in the form of triangular surfaces (stereolithographic files: STL). These models were manually divided into four semantic classes: the vertebral body, the left transverse with the left superior articular processes, the right transverse with the right superior articular processes, and the spinous process with the lamina (Figure 2C). The division was arbitrarily chosen to achieve satisfying registration of the template model prior to template mesh-fitting. The rationales behind the semantic segmentation step, and the consequent ability to select patient-specific anatomical landmarks, are multiple. Since the same landmarks were labeled on the SSMs, the transformation to place the deformable models can be found automatically. Additionally, initializing the non-rigid registration using landmarks has been shown to improve results (Clogenson et al., 2015). Since the training of the network needed each vertebral model within the dataset to have the same number of points (equal to N in Figure 3), the segmented vertebrae were preprocessed to prepare the training dataset. The complete set of vertebral 3D models was down- or up-sampled before the semantic division to match a defined number of points. To assure homogeneous meshes and an accurate representation of details, N was set to 2,947, with 4,000 triangular faces in each mesh. The final training dataset comprised 138 vertebral meshes that were manually segmented and semantically divided.

##### Training process

Regional information on the segmented meshes was needed to initialize the position of the template models. The Tensorflow Graphics (TG) framework uses revised CNNs to segment 3D models in semantic parts (Valentin et al., 2019), the network is referred as graph convolutional network and is able to process point cloud data (Verma et al., 2018). A simplified CNN version of the FeaStNet architecture was implemented in TG according to Valentin et al. (2019) and used to semantically divide the segmented models of the vertebrae into four parts. Figure 3 shows the network structure: first, each vertex of the point was encoded by a mesh encoder into a N × 4 logits, where N is the number of points and 4 the number of semantic classes. The mesh encoder consists in 1 × 1 convolutions linear layer to change the input dimensions, a sequence of feature steered graph convolutions was then followed by 1 × 1 convolutions to convert the output to a N × 4 logits. The training was run using the Adam optimizer with ε = 1e – 8 and a learning rate equal to 0.001 (Valentin et al., 2019). The output of the mesh encoder was used to perform a soft-assignment of each vertex to one of the four classes (Verma et al., 2018).

The trained network was used to establish correspondence between vertebral 3D models according to the steps depicted in Figure 2. The division into semantic classes facilitated positioning of the template SSMs by the automatic identification of labeled anatomical locations. The landmarks were found by computing the center of mass of each semantic part, which was then projected on the surface mesh. A rigid transformation was defined using the identified landmarks on the 3D models and the corresponding points labeled on the SSMs. To account for the uncertainty in the identification of anatomical points, the iterative closest point method (Besl and McKay, 1992) was used to fine-tune the final position, providing an improved initialization for the morphing of the SSMs in the subsequent step.

#### Template Model’s Fitting

Five SSMs were created, one for each lumbar vertebra. Each model was trained using manually segmented 3D meshes of lumbar vertebrae, which were not extracted from the images used for the training of the CNNs. The vertebral meshes were divided based on the spinal level and multiple training datasets were created consisting of 100 meshes pro lumbar level. The SSMs were built using a Procrustes Alignment to align each dataset, which was followed by a non-rigid registration as investigated in Clogenson et al. (2015). The resulting 3D models are used to construct the SSMs by finding the main shape variations with a principal component analysis (PCA). The manual creation of the reference meshes used as templates assures a smooth surface and a homogeneous triangularization of elements along the whole vertebral models. The preparation of the template, the training of the SSMs, and the registration framework were implemented in the Scalismo package (University of Basel, Switzerland) (Lüthi et al., 2012). The semantic division together with the positions of the 4 landmarks identified by the trained FeaStNet network provided an optimal initialization for the non-rigid registration of the template SSMs. The non-rigid registration was implemented according to Clogenson et al. (2015) and is based on a point set to image registration. The registrations of the SSMs were further constrained by the four landmarks which were identified on the templates in advance. An additional step was added to the framework presented in Clogenson et al. (2015) and consists in a projection of the SSMs points along the mesh’s normal vectors toward the target surfaces. The correspondence of the landmarks together with the projection step increased the performance of the non-rigid registration as well as the precision of correspondence between registered vertebral structures. Constraining the registration allowed a reduction of the search space, leading to a more robust and faster registration process (Clogenson et al., 2015). To finalize the non-rigid registration, a projection of the template nodes was performed after the last iteration along the model’s normal vectors on the target triangulated model resulting from the DenseVNet segmentation. This step enables the union of the correspondence property of SSMs with the precision of DL segmentation. The resulting patient-specific anatomical models have the same triangularization properties as the reference meshes of the SSMs but represent the patient’s spinal structures.

#### Anatomical Model Evaluation

To evaluate the anatomical models, the training of the DenseVNet network was performed using 47 labeled 3D CT images while five were excluded for inference. Ten-fold cross-validation was performed to assess the performance of the anatomical model generation. During each iteration, the network was trained anew and tested on the five excluded images (containing three vertebrae each). Thus, 150 (10 × 5 × 3) vertebral 3D models were automatically segmented and compared to manually segmented 3D models (ground truth).

The generation of the anatomical model was evaluated directly after the DenseVNet segmentation and after the non-rigid registration of the deformable model. This enables tracking of the segmentation performance throughout the pipeline. Both outputs were compared to the ground truth vertebrae. The evaluation criteria were: the DC, the mean surface distance (MSD), and the Hausdorff distance (HD).

### Finite Element Modeling and Simulation

#### Model Validation Using Cross-Validation Healthy Data

**FIGURE 4:**
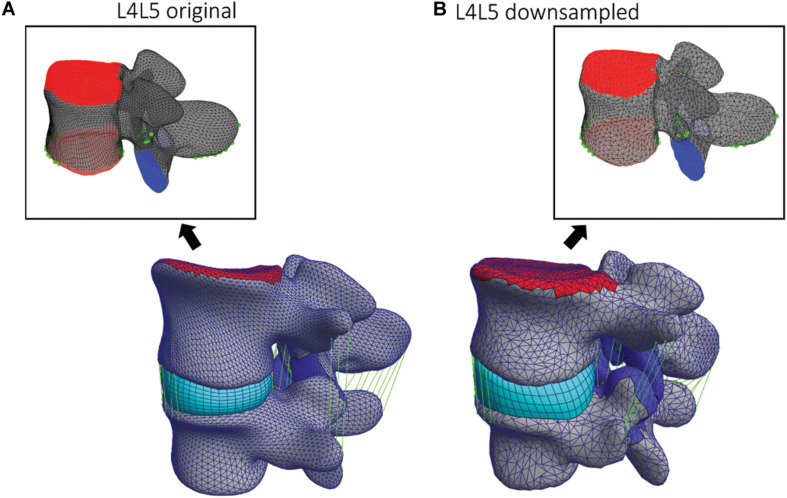
**(A)** Original and **(B)** down-sampled template L4 models and corresponding L4L5 FSU FE models. On the two template models, the facets and endplates surfaces are labeled (blue and red) as well as the ligaments attachment points and paths (green).

The five images segmented in the last iteration of the cross-validation were used to create and run FE simulations of all L3L4 and L4L5 FSUs of each patient, respectively. The dimensions of the resulting healthy vertebrae were on average: 86.28 mm left-right, 88.43 mm anterior-posterior, and 48.35 mm inferior superior. The registration of the deformable templates ensures the correspondence of nodes across the fitted surface models (Verma et al., 2018; Wu et al., 2019). The correspondence property allows to label essential regions for the creation of the final FE model. The facet joints, the vertebral endplates, and the ligament attachment points were manually labeled on the template of each vertebra *a priori*, as shown in Figure 4 for a model of L4, and used to define contacts and boundary conditions. The registered SSM resulted in patient-specific surface meshes allowing the personalization of insertion points and contact surfaces, adjusted according to the patient anatomies by the registration step. The resulting surface models were converted into volumetric meshes and divided into cortical and trabecular bone. The inter-subject variability of the material properties was not considered. All the material properties of the FE model were implemented according to Finley et al. (2018). The trabecular bone, the IVD nucleus, the vertebral endplates, and the facet cartilage were represented using a neo-Hookean model; the cortical bone is modeled as orthotropic elastic material, and the superior endplate of the upper vertebra (indicated in red in Figure 4) was rigidly modeled and used to apply the pure moment loading. To represent the annulus, a compressible Holmes-Mow material model was coupled with two fiber components. The material properties of the FE models are summarized in Table 1.

**TABLE 1:** Material properties used for the FE simulations of the healthy FSUs.

Structure	Material model	Young’s modulus (MPa)	Poisson’s ratio
Cortical bone	Orthotropic elastic	E_1_ = 8,000	v_12_ = 0.4
		E_2_ = 8,000	v_23_ = 0.3
		E_3_ = 12,000	v_31_ = 0.35
Trabecular bone	Neo-Hookean	E = 100	v = 0.2
Vertebral endplate	Neo-Hookean	E = 1,000	v = 0.3
Nucleus pulposus	Neo-Hookean	E = 1	v = 0.49
Annulus matrix	Holmes-Mow	E = 1	v = 0.4
		β = 3.4	
Annulus fibers	Fiber-exponential-power	α = 65	–
		β = 2	
		ξ = 0.296	
Facet cartilage	Neo-Hookean	E = 30	v = 0.4

The anterior longitudinal ligament, posterior longitudinal ligament, supraspinous ligament, intertransverse ligament, ligamentum flavum, and interspinous ligament were modeled as non-linear and tension-only elements (Finley et al., 2018). The endplates of the superior and inferior vertebrae served as reference to place and fit the IVD. The superior endplate of the upper vertebra was used to apply the pure moment of 7.5 Nm in various directions to simulate flexion, extension, axial rotation, and lateral bending, whereas the inferior endplate remained constrained in all the degrees of freedom (DOF). Contact areas of the FJs were defined in facet’s cartilage regions and implemented as sliding interfaces enforcing a non-penetration constraint. Figure 4 shows how those regions and landmarks are marked on different template models. Using the endplates’ nodes, a hexahedral mesh defining the IVD was created for each FSU and deformed to assure a tied contact with superior and inferior vertebrae. These steps defining the FSU FE model were implemented in the GIBBON package (Moerman, 2018) and the simulations were performed within the open-source tool FEBio 2.9 (Maas et al., 2012) using an implicit FE solver. The pipeline was implemented such that the creation of the FE models and the corresponding simulations were automatically run in sequence. In this study, only the geometrical inter-subject differences were considered; because automating the creation of FE models in a single pipeline was the primary goal.

For the simulation of flexion, extension, lateral bending, and axial rotation movements all the DOF of the inferior endplate were constrained and a pure moment of 7.5 Nm was applied to the superior endplate of the upper vertebra. To test the mesh convergence of the FE simulations, down-sampled deformable models (Figure 4B) were registered to the L4 and L5 vertebrae of one subject. The resulting range of motion (ROM) from axial rotation simulations was compared among the different down-sampled models to assess mesh-convergence of the FE simulations and to compare the results using different template models.

#### Evaluation on Pathological FSUs

The whole pipeline was further evaluated on 5 pathological cases, in addition to the five non-pathological cases from the training dataset. The 3D CT images were initially acquired as part of a previous study in our institution (Widmer et al., 2020a) with approval from local ethical authorities (BASEC Nr. 2017-00874). The specimens selected for this project were excluded in Widmer et al. (2020a) as they were classified as severely pathological by a medical professional. To evaluate the robustness of the pipeline, different lumbar spinal segments were included. The five pathological FSUs were composed of two L2L3, two L3L4, and one L1L2. The anatomical dimensions of the pathological vertebrae were similar to the ones of the healthy FSUs: 87.37 mm left-right, 95.9 mm anterior-posterior, and 53.3 mm inferior-superior. The pathological specimens originated from fresh frozen cadavers (Table 2). The classification of IVD degeneration was performed by Pfirrmann grade (Pfirrmann et al., 2001) based on the segmented 3D models, the CT, and the MR images. From the five pathological lumbar segments, 3 had Pfirrmann grade equal to 4, and in two cases the specimen was classified with a Pfirrmann grade equal to 5. The Weishaupt grade (Zhou et al., 2016) for FJ degeneration in the 5 pathological FSUs was between 2 (narrowing of the facet joint space), and 3 (narrowing of the facet joint space and/or moderate osteophytes, and/or moderate hypertrophy of the articular process, and/or mild subarticular bone erosions). In this study, the vertebral structures were segmented and fitted using the trained neural networks and the statistically deformable templates as described above, but the IVD was fitted between the labeled upper and lower vertebral endplates. The accuracy in the segmentation of the bony structures defined the shape of the IVD mesh that was enforced to be in contact with the vertebral endplates. To account for the existing pathology, the material parameters were corrected automatically. For the FSUs with Pfirrmann grade 4, the Young’s modulus values of the nucleus pulposus and of the annulus matrix were changed to 1.4 and 4.5 MPa, respectively. The Poisson’s ratio was changed to 0.42 in the nucleus pulposus. The remaining properties were as stated in Table 1. For Pfirrmann grade 5, the Young’s modulus of the nucleus pulposus and the annulus matrix were set to 2.2 and 5.5 MPa, respectively. The Poisson’s ratio of the nucleus pulposus was changed to 0.32 (Wang et al., 2012). The altered material properties reflect a stiffening of the IVD concomitant with a loss of fluid content as the degeneration progresses (Wang et al., 2012).

**TABLE 2:** Demographics and degeneration state for the five pathological samples.

Specimen	Sex	Age (y)	Height (cm)	Weight (kg)	Pfirrmann
S182452	Male	62	173	79	4
S182664	Male	75	185	98	5
S181997	Male	82	185	91	4
S182571	Female	84	165	67	4
S181934	Male	75	188	79	5

Although material properties mapped from imaging data would be desirable, patient-specific material properties have not been included in this study. The main objective of this work is the automated generation of anatomically accurate FSUs models. Any material mapping method could then be implemented on these models. Nevertheless, we used our models to simulate ROM and evaluated the reasonability of results by comparing them to reported values from the literature.

## Results

### Cross-Validation

We report the results of the cross-validation in terms of the segmentation resulting from the trained DenseVNet network, and precision of the 3D model after landmark-based template model fitting (section “Segmentation”) corresponding to the 150 healthy vertebrae. The FE simulations’ results are presented for the 10 FSUs resulting from the last iteration of the cross-validation (section “Finite Element Modeling”). The segmentation metrics before and after the deformation of the template model showed a slight decrease in performance in terms of DC, on the other hand, the MSD and the HD were better after the template model deformation.

#### Segmentation

The evaluated metrics were computed for each vertebra in the 5 excluded images segmented during cross-validation. For each iteration, the metric values were averaged over the five images for each vertebral level. Table 3 summarizes the average of the evaluation metrics for all the 3 vertebrae after 10 iterations of the cross-validation (*N* = 50 per vertebral level). The resulting metrics show how the trained network achieved state-of-the-art performance in the segmentation of lumbar vertebrae with average DC equal to 93.71%. The MSD was equal to 0.88 mm and the HD to 11.16 mm, on average among all the three vertebrae for all the five images excluded in each iteration of the cross-validation (*N* = 150, combining the three lumbar vertebrae). Table 3A shows the same metrics divided per level. After the non-rigid registration step, which was performed directly after each iteration of the cross-validation, the DC performance decreased by 3.05%, but the MSD and HD performances increased by 23.86 and 34.23%, respectively (Table 3B). The non-rigid registration of the template models acts as a smoothing filter on the segmented vertebrae, lowering the overall performance in terms of the DC, but improving the surface distance metrics of the segmented models by filtering out large HD values.

**TABLE 3:** Mesh metrics from the cross-validation.

	(A) DenseVNet segmentation			(B) Non-rigid registration		
	DC (%)	AD (mm)	HD (mm)	DC (%)	AD (mm)	HD (mm)
L3	93.71 ± 8.2	1.02 ± 1.71	11.9 ± 11.61	92.54 ± 1.8	0.55 ± 0.14	6.39 ± 3.42
L4	93.68 ± 5.9	0.90 ± 1.24	11.1 ± 9.4	90.62 ± 2.5	0.70 ± 0.21	7.27 ± 2.51
L5	93.73 ± 4.9	0.72 ± 0.77	10.4 ± 7.03	89.38 ± 5.7	0.75 ± 0.33	8.30 ± 4.82

*The performance is evaluated directly after the DenseVNet Segmentation **(A)** and after the registration of the deformable models **(B)**. Each metric was evaluated on 50 vertebral models, for each lumbar level.*

#### Finite Element Modeling

**FIGURE 5:**
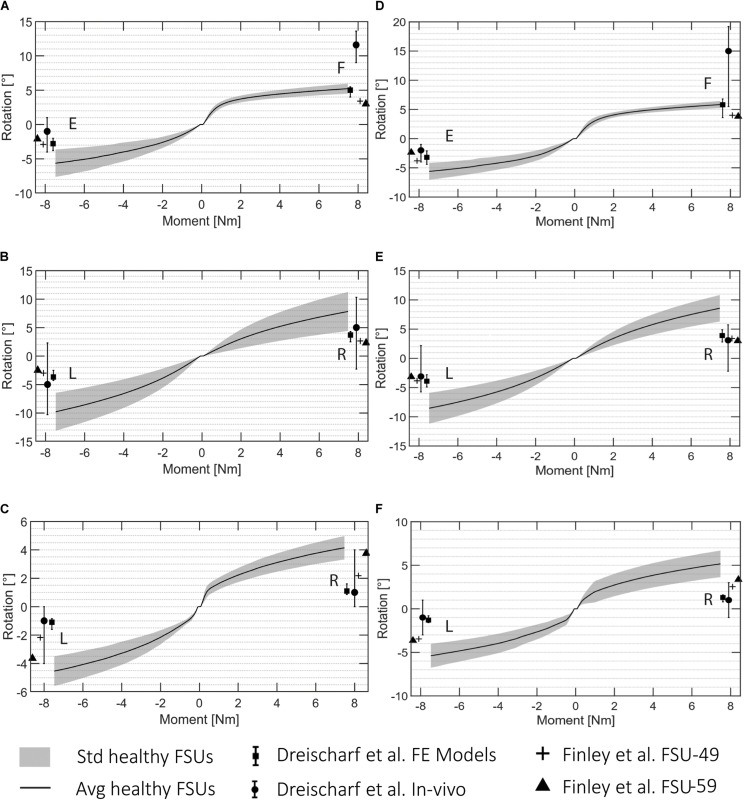
ROM data for all the automatically created FSU models for L3L4 **(A–C)** and L4L5 **(D–F)**. **(A,D)** flexion (F)/extension (E), **(B,E)** lateral bending (R: right, L: left), and **(C,F)** axial rotation (R, L). The standard deviation is shown in gray and the average rotation in black. The literature values presented in Dreischarf et al. (2014) are visualized for the *in vivo* and *in silico* results, and the reported values from Finley et al. (2018) are presented for a FSU FE model of a 49 years old patient (FSU-49) and 59 years old patient (FSU-59).

The FE simulations provide load-deformation behavior for flexion, extension, lateral bending, and axial rotation. The ROM values averaged over all the 5 subjects are shown in Figure 5 for each type of motion and two lumbar FSUs.

These ROM values are depicted with reported values for single FSU models from two different studies (Dreischarf et al., 2014; Finley et al., 2018). Figure 5 shows the average and the standard deviation of the intersegmental rotation angles vs. the change in the applied moment from −7.5 to 7.5 Nm. The results were in agreement with those from Finley et al. (2018) and with the reported values from the six computational models analyzed in Dreischarf et al. (2014). The ROMs resulting from the FE simulations in this study were 4.49°–6.45° and 3.64°–7.64° for flexion and extension, respectively, 4.39°–13.12° for lateral bending, and 3.31°–6.75° for axial rotation. The ranges of the flexion and extension angles obtained in Finley et al. (2018) were 3°–4° and 2.1°–3.8°, respectively, whereas the ranges for lateral bending and axial rotation were 2.3°–3.84° and 2.18°–3.75°, respectively. The ROMs reported in Dreischarf et al. (2014) from median *in vivo* values differed more to the simulated ones, they reported angles between 5.5° and 19.2° and −1° and 4° for flexion and extension, respectively. The ranges for lateral bending and axial rotation were independent of the rotational direction and equal to −2.3° and 10.3° and −1° and 4°, respectively.

**FIGURE 6:**
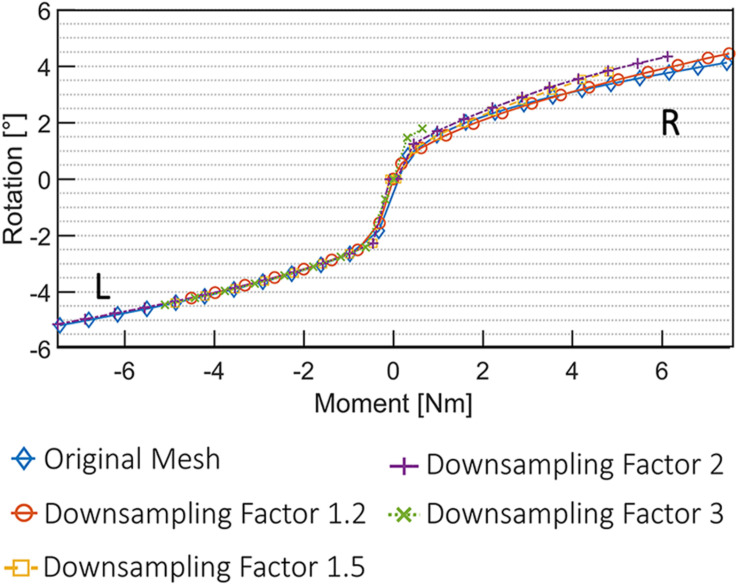
Convergence analysis using five different 3D deformable models. An FSU of L4L5 was used for the simulation of axial rotational movement (R, right; L, left).

The results of the mesh convergence analysis performed on an L4L5 FSU of one subject are summarized in Figure 6. The reference meshes of the SSMs representing the two vertebral levels were down-sampled by factors of 1.2, 1.5, 2, and 3 and registered to the DenseVNet outputs using the same landmark-based fitting method. The down-sampled models did not affect the non-rigid registration precision since it was constrained by the same landmarks identified using the FeaStNet network and were able to achieve patient-specific geometries. Large down-sampling factors lead to early non-convergence of the simulations, likely related to the adverse effect of large element size in contact modeling. As the original mesh size offers a good compromise between registration precision and computational costs, all following models were created with this mesh size.

### Pathological FSUs

**FIGURE 7:**
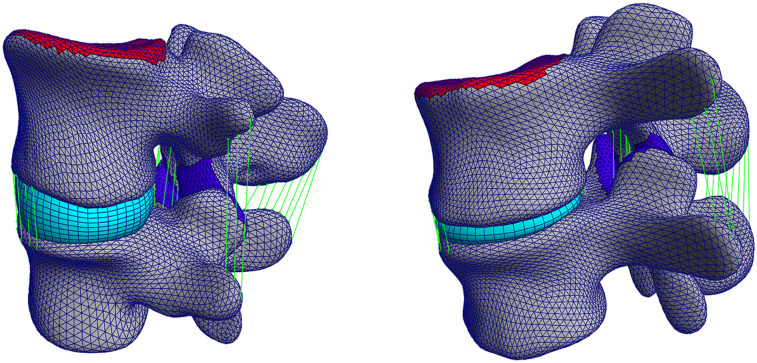
Left: a healthy FSU FE model evaluated in the cross-validation, right: a pathological FSU FE model with Pfirrmann grade equal to 5.

The pipeline was also evaluated on pathological 3D CT images and the same metrics were computed after the segmentation of the five pathological FSUs. The results show how the performance of the pipeline resulting from the cross-validation translates to clinically relevant cases. After segmentation, the average DC was equal to 90.4 ± 2.9%, the MSD was 0.66 ± 0.1 mm, and the average HD was 10.7 ± 4.4 mm. Figure 7 shows a comparison between a healthy FSU FE model from the cross-validation and a pathological FSU with Pfirrmann grade equal to 5. The vertebral structures were segmented and fitted with the deformable templates as described above, but the elements of the IVD were reduced automatically according to the mean distance between the labeled endplates.

**FIGURE 8:**
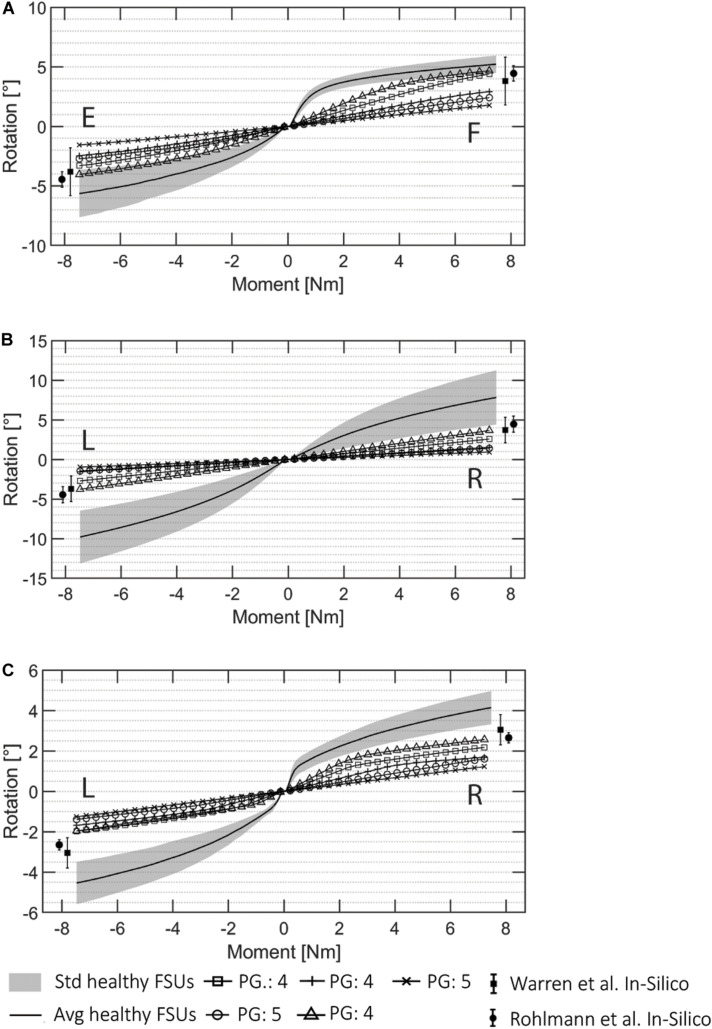
ROM data for all the automatically created pathological FSU models for **(A)** flexion (F)/extension (E), **(B)** lateral bending (R: right, L: left), and **(C)** axial rotation (R, L). The Pfirrmann grade (PG) of each FSUs is marked in the image. The pathological results from the literature presented in Rohlmann et al. (2006) and Warren et al. (2020) are visualized.

The FE simulations of the pathological cases are presented in Figure 8 together with the ROMs of the healthy FSUs for flexion/extension, lateral bending, and axial rotation. The ROM values for pathological FSUs presented in two different studies (Rohlmann et al., 2006; Warren et al., 2020) are included in Figure 8. The resulting rotations decreased with the same moment of 7.5 Nm applied. From the FE simulations, a difference in intersegmental rotation between specimens presenting Pfirrmann grade equal to 4 and 5 was noticeable.

The ranges resulting from the pathological simulations were on average 57.7% lower than the average angles resulted for the same simulations on the healthy FSUs. The flexion/extension simulations resulted in ranges of 1.79°–4.67°, and 1.56°–4.04°, respectively. Lateral bending was between 0.93°–3.76° in left and right directions, and axial rotation resulted in movements between 1.22° and 2.56° in left and right directions. The ROMs decreased with respect to the healthy ones according to the degeneration grades assigned during the classifications (Widmer et al., 2020a) the average ROM reduction for FSUs classified as Pfirrmann equal to 4 was 49.3%, and the reduction for FSU with Pfirrmann grade equal to 5 was 70.4%. Therefore, FSUs with a higher degenerative condition corresponded to a reduced ROM from the FE simulations. This was true for all the three simulated motions. Because of the asymmetrical FJs and/or IVD degenerations, lateral bending and axial rotation simulations resulted in different movements for the left and right sides.

## Discussion

The presented pipeline combines deep learning methods to perform image and semantic mesh segmentations, together with FE modeling to automatically generate and analyze patient-specific FSUs. The segmentation of vertebrae using the DenseVNet network produced highly accurate results comparable to the state-of-the-art automated segmentation methods (Sekuboyina et al., 2017; Janssens et al., 2018; Vania et al., 2019) for both healthy and pathological FSUs. The simulations using the open-source FE solver FEBio during cross-validation had comparable results to the ones reported in the literature (Dreischarf et al., 2014; Finley et al., 2018) and also for the simulated pathological cases (Rohlmann et al., 2006; Jiang and Li, 2019). The whole pipeline is based on a cropped 3D CT image of lumbar spinal segments of interest and does not require any other inputs or manual interaction to perform a biomechanical analysis of FSUs. The current time required to create a FE model of the spine is rarely reported but the state-of-the-art process includes using a software for the segmentation of volumetric images to obtain surface meshes. The latter are again imported in a second software to create volumetric models and perform the FE simulations including pre- and postprocessing steps (Bah et al., 2009; Haj-Ali et al., 2019; Jiang and Li, 2019; Özmen and Günay, 2019). From our empirical experience, the process of segmentation, meshing, FE model preparation and simulation can take up to several days. In addition, since many tedious manual steps are needed for the model’s preparation, the robustness of the process could be affected. However, replicability is key when different FSU configurations have to be tested, to identify clinically relevant differences between patients’ structures. With the proposed pipeline, we were able to simulate simple movements for many FSUs from a 3D CT image with a minimal amount of user interaction for cropping the input image, requiring a time effort of about 30 s. Both segmentation and FE simulations were evaluated on five healthy cases within the cross-validation, and on five pathological FSUs selected from a different dataset of images. The entire pipeline, from image cropping to the patient-specific biomechanical results for the FSUs of interest, is about 2 h. Compared to current state-of-the-art this represents a significant reduction in time and manual interaction.

The resulting ROMs of the healthy FSUs were within the range of other published FE models but slightly outside the range of *in vivo* measured ROMs (Dreischarf et al., 2014). The comparison between models is difficult since the patient-specific geometries are potentially affecting the ROM. The geometrical variability was mostly not considered in FE modeling, but different structures could lead to different ROM results. Patient-specific material properties were neither considered in the creation of the FE model, potentially introducing further deviation from *in vivo* behavior (Dreischarf et al., 2014). From the obtained results, we can observe the larger influence of the facet joints in the lateral bending and axial rotation simulations due to specificity of patient geometry when estimating joint motion. However, the different results between healthy and pathological cases were verified and the pipeline is able to capture different degenerated states automatically, as shown in Figure 8. The resulting ROMs obtained for the pathological cases were in line with the clinical degeneration grades, correctly showing a reduction in the intersegmental rotation angles in comparison to the healthy FSUs ROMs. Furthermore, the simulated ROMs partially agreed with the pathological values reported in the *in silico* studies from Rohlmann et al. (2006) and Warren et al. (2020). However, a direct comparison of values may be compromised due to simplified FE models in those studies.

The automatization of simulations for the proposed pathological cases may accelerate the inclusion of FE simulations in the planning of spinal surgery. It allows studying how different degrees of degeneration affect the FSU’s motion patient-specifically. A biomechanically based preoperative assessment is needed for patients presenting signs of IVD and/or FJ degeneration, to obtain an optimal patient-specific surgical plan taking pre-existing conditions into account (Li et al., 2015; Perolat et al., 2018) and to customize corresponding patient treatment (Zhou et al., 2016). The selection of an FSU of interest is the only manual step within the suggested pipeline, making its integration into the surgical planning workflow easier.

Following, we discuss some of the limitations of this work. The implemented pipeline was not evaluated on publicly available datasets. However, it achieved state-of-the-art precision employing standard medical data. Our goal was not to outperform existing segmentation methods, but rather to combine different solutions for both segmentation and FE modeling tasks in a single automated pipeline. Biomechanical analysis has the potential to improve surgical planning but its integration in the clinical workflow is essential. Hence, the acceleration of the time-consuming preparation of FE models was targeted using the proposed implementation. The input images must still be manually cropped by a user, yet this could be considered as a user control point to select the correct levels of interest. The cropping itself represents a very quick step for medical professionals and the pipeline functions with different cropping sizes and regions of the lumbar spine. This is one of the main differences to other semi-automatic approaches consisting of labor-intensive (and therefore costly) and time-consuming steps (Zadpoor and Weinans, 2015; Haj-Ali et al., 2019; Nikkhoo et al., 2020). Indeed, many training data used in this work were manually annotated, for example the segmentation of 3D CT images, or the anatomical landmarks defined on the reference meshes of the SSMs. Although manual annotation may be a possible insertion of errors, it is also comparable with the state-of-the-art creation of biomechanical models from CT data. We believe that the advantages are multiple and as a result of the different trainings, our pipeline is able to reduce inter-user variability which is normally intrinsic in the creation of biomechanical models mostly due to the inevitable involvement of manual work. The automation of multiple steps has the natural consequence of improving consistency and allowing comparisons between multiple patient-specific analysis. In addition, new training datasets may be created with a drastic decrease in manual efforts.

As another limitation, patient-specific material mapping was not implemented. This could be critical for a correct patient-specific model, since the inclusion of patient-specific material properties plays a crucial role in generating clinically relevant outputs. The lack of patient-specific material properties may influence the resulting ROMs. Different studies have shown how much biomechanical parameters vary between subjects (Van Rijsbergen et al., 2018; Sawa et al., 2020; Wawrose et al., 2020) and how the ROM is affected by these variations. The ROMs reported in this work were partially out of range as compared to other studies (Dreischarf et al., 2014). Also, minor inaccuracies in the vertebral geometry, and especially in the FJs, may influence the ROMs (O’Reilly and Whyne, 2008). In our pipeline, small errors resulting from the automated segmentation were predominantly concentrated in the FJ regions. On one hand, this has a negative impact on the performance metrics of the segmentation, yet a small one since the surface of the FJ regions are small as compared to the overall surface of a vertebra. On the other hand, this could further lead to penetrations between the vertebral meshes of different levels after SSM registration. In such cases the FJ gap is created automatically by making fine adjustments to mesh regions. The resulting small morphological deviations from the real patient’s anatomy may have a significant effect on simulated motion, and are a potential explanation for the large ROM values observed in lateral bending and axial rotation. Particularly in these modes of spinal movement facet joints act to prevent from excessive motion, which may have been compromised on the current study. In addition to ROM, the FJ forces and intradiscal pressure may deliver insight on the validity of the presented model, and we intend to further improve the model by also evaluating these measures. In any case, a comprehensive validation of the desired outputs is required prior to any clinical application, as with any computational model.

The intent of this study, however, was to prove that a complete automated concept for the biomechanical analysis of lumbar 3D CT images is possible in a single pipeline. Our main objective was the implementation of a complete automated pipeline able to generate and simulate anatomically accurate FE models, also in pathological conditions. More complex FE models could contain patient-specific material mapping and implants could be also added. The improvement of the patient-specificity of the model using material mapping, together with an improved version of the material models in the FE simulations, are the next expected steps to improve our pipeline (Widmer et al., 2020a,b). Additionally, the identification of clinically relevant FSUs could be implemented automatically using existing techniques for image cropping, vertebrae localization (Chu et al., 2015; Korez et al., 2015; Sekuboyina et al., 2017), and segmentation of pathological (Ibragimov et al., 2017) or fractured vertebral structures (Roth et al., 2016) including the segmentation of the IVD (Zheng et al., 2017; Han et al., 2018). In its current implementation, the pipeline is not able to run the FE simulations for pathologies such as fused vertebrae or disc prolapse. An enlarged or additional training dataset will be needed to allow the accurate segmentation of pathological cases. These may then also include fractured or collapsed vertebral structures. The current pipeline is limited in that it is only able to create anatomical models of intact vertebral structures without osteophytes. The automatic identification and segmentation of such cases avoiding *biases* is one of the bottlenecks in medical images processing (Galbusera et al., 2019). However, in our group, the accelerated creation of anatomically accurate models has supported the preparation of instrumented FE models to investigate the bone-screw interface (Widmer et al., 2020b). Even if the current pipeline only accepts very specific types of deformities, it represents a first successful attempt for the automation of biomechanical analysis. The time saved for model preparation enables computational analysis at a low cost, which we believe is an important step toward their clinical integration.

## Conclusion

The results obtained from the implemented pipeline demonstrate a novel and powerful approach for automatic generation of predictive models with results that are comparable to manually segmented and manually generated FE models, in both healthy and pathological FSUs. The approach reduces manual interaction to a minimum, involving only the cropping of the 3D CT image as input to the pipeline for fast generation of anatomically accurate FE models. The automatization of the labor-intensive steps of vertebrae segmentation, landmark identification, and finite element model generation reduces clock time by orders of magnitude as compared to manual preparation. Results of FE simulations are available in about 2 h from feeding cropped images into the pipeline. Notably, the approach allows modeling of pre-existing pathological conditions in an automatic fashion. The advances described in this work are a first step toward enabling substantial improvements for computer-assisted surgical planning of the spine thanks to the integration of patient-specific biomechanical analysis.

## Data Availability Statement

The data analyzed in this study is subject to the following licenses/restrictions: The dataset is under property of a third party. Requests to access these datasets should be directed to MF, Mazda.Farshad@balgrist.ch.

## Ethics Statement

The studies involving human participants were reviewed and approved by ID: BASEC: 2019-00698. Written informed consent for participation was not required for this study in accordance with the national legislation and the institutional requirements.

## Author Contributions

SC developed the pipeline and was responsible for the technical details and designed the training and validation of the different CNNs and the implementation of the statistical models and analyzed the FE simulations results and wrote the manuscript and prepared the figures. FC contributed to the design of the models and to the presentation of the results and assisted with the simulations and supported the figures’ creation and also contributed to the final manuscript. JS supervised the project and contributed to the interpretation and presentation of the results. JS, MF, and MS contributed to the final version of the manuscript. MF supervised the design of the research and contributed to the interpretation of the results from the clinical side. MS supervised the project and technical details and designed the structure of the pipeline and supported SC with the analysis of the results and the preparation of the figures. All authors contributed to the article and approved the submitted version.

## Conflict of Interest

The authors disclose ties to Incremed AG, Zurich, Switzerland, which is developing solutions for the preoperative planning of spinal surgeries. MF was a board member and SC was an employee of Incremed AG.

## Abbreviations

CT: Computed Tomography
CNN: Convolutional neural network
DOF: Degree of freedom
DC: Dice coefficient
FJ: Facet joint
FE: Finite element
FSU: Functional spine unit
HD: Hausdorff distance
IVD: Intervertebral disc
MSD: Mean surface distance
ROM: Range of motion
3D: Three-dimensional.
